# Ovarian Pregnancy following Intracytoplasmic Sperm Injection and Embryo Transfer: A Case Report

**DOI:** 10.1155/2012/389107

**Published:** 2012-06-17

**Authors:** Amar Ramachandran, Swati Sharma, Kumar Pratap, Bhakta Rajesh, Vasudeva Akhila, Akhila Ramayapally, Manna Valiathan

**Affiliations:** ^1^Department of OBG, Kasturba Medical College, Manipal 576104, India; ^2^Department of Pathology, Kasturba Medical College, Manipal 576104, India

## Abstract

Ovarian pregnancy is a rare form of ectopic pregnancy following ICSI-ET, and the diagnosis depends on the physicians suspicion and experience. Preservation of ovarian tissue during surgery is of utmost importance to preserve fertility. We present a case of ovarian pregnancy who had a successful treatment preserving the ovary.

## 1. Introduction

The incidence of an ectopic pregnancy varies in a range between 0.3% and 1.4% of all pregnancies [1]. An increased incidence of ectopic pregnancies up to 5-6% after in vitro fertilization (IVF) and embryo-transfer (ET) is a well-known phenomenon. However, primary ovarian pregnancy is a rare phenomenon following natural conception or IVF-ET. The incidence of primary ovarian pregnancy is 3.3% of all ectopic pregnancies [2], and the incidence reported following IVF-ET is 0.27% per clinical pregnancy [3]. We report a case of primary ovarian pregnancy following ICSI for primary infertility treatment. The case presented here is interesting in terms of both the rarity of this entity and the successful therapy preserving the ovary.

## 2. Case Report

**Figure 1:**
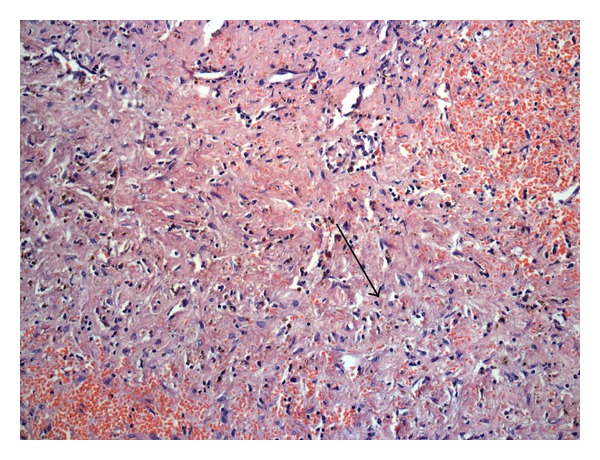
Ovarian stroma H&E × 100.

**Figure 2:**
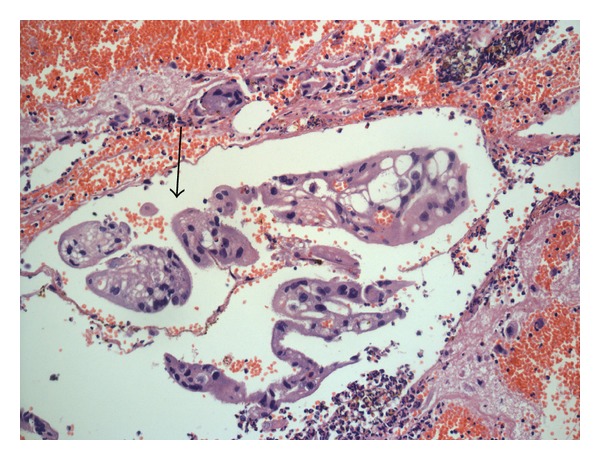
Trophoblast in hemorrhagic stroma. H&E × 100.

She was 33 years, and married for 4.5 years. She underwent ICSI for male factor infertility. Serum *β*-hcg on the fourteenth day of embryo transfer (ET) was 39 mIU/mL. She had spotting per vagina 5 days later (19th day of ET), and the serum *β*-hcg was 118.1 mIU/mL. The vaginal sonography did not reveal an intrauterine or extrauterine gestational sac, and she was followed up. On the 30th day of ET (6 weeks period of gestation), she presented to the hospital with complains of pain abdomen for one day and gradual distension of the abdomen. Clinically, she was pale, but the vital signs were stable. Vaginal sonography showed uterine cavity empty, right adnexa had a hypoechoic area of 2.5 cm × 2.1 cm, and the left adnexa was normal. There was significant fluid in the pouch of Douglas. Serum *β*-hcg was not repeated at that time. Emergency laparotomy was performed with the diagnosis of ectopic pregnancy. 300 mL of blood and 150 gm of clot were seen in the peritoneal cavity. Right ovary was found ruptured and bleeding actively, and hence right side partial ovariectomy was performed and sutured. Both the fallopian tubes and left ovary were normal. The pathologic examination confirmed the clinical diagnosis (Figures 1 and 2). She had ICSI twice later and oocytes were retrieved from right ovary too. She conceived in the second attempt and delivered a male child.

## 3. Discussion 

Primary ovarian pregnancy is an uncommon form of ectopic pregnancy. Marcus and Brinsden reported an incidence of 6% following IVF-ET (8 primary ovarian pregnancies following 135 ectopic pregnancies after IVF-ET) [4]. But the incidence following natural conception is much lower, ranging from 1/16,000 to 1/1,500 deliveries accounting for 3.3% of all ectopic pregnancies.

The four Spiegelberg's criteria for ovarian ectopic gestation are: (1) fallopian tubes including fimbria must be intact and separate from the ovary; (2) the pregnancy must occupy the normal position of the ovary; (3) the ovary must be attached to the uterus through the utero-ovarian ligament; and (4) there must be ovarian tissue attached to the pregnancy in the specimen [5].

Diagnosis of primary ovarian pregnancy is very difficult because of the rarity and the asymptomatic nature before rupture. Although the combination of serum *β*-hcg and vaginal sonography increases the diagnostic accuracy of ectopic pregnancy, the diagnosis of primary ovarian pregnancy depends on the physicians suspicion and experience. As a rule, if the serum *β*-hcg level is >1000 mIU/mL without an intrauterine gestational sac by vaginal sonography, patient is regarded to have ectopic pregnancy. 

Fertilisation of the ovum inside the ovary or implantation of the fertilised ovum in the ovary seems to be most responsible causes in the etiology and pathogenesis of ovarian ectopic pregnancy. In the case we described, the exact mechanism of ovarian pregnancy after ICSI was unclear since there were no predisposing factors for ectopic pregnancy except that four embryos have been transferred in our case. One of the most likely probabilities is reverse migration of one of these embryos toward the fallopian tube and implantation in the ovary [6]. This unusual event could be a result of the volume and pressure of culture medium injected during embryo transfer. Another contributory factor in the pathogenesis might be head tilted down position given to the patient following ET. Knutzen introduced the reverse migration concept and demonstrated the fate of embryos “in utero” by observing that radiopaque dye could enter the fallopian tubes in 38.2% of the patients easily after a mock ET [7]. In our patient, a tilted position was given following ET and 10–15 *μ*L of the media was used and the transfer was done under ultrasound guidance. Difficult ET may be another possible factor due to resulting in additional stimulation of junctional zone contractions which increases the risk of ectopic pregnancy. Lesny et al. showed that a difficult ET stimulates junctional zone contractions and that strong endometrial waves in the fundal area of uterus could move mock embryos into the fallopian tubes. They also noted that manipulation with tissue forceps, for the purpose of facilitating ET, could affect uterine contractility [8] but such events were not encountered at the time of ET in this patient. Reverse migration of an embryo also could be due to high estrogen levels after ovarian stimulation [9]. In our case, serum estradiol concentration was 4300 pg/mL on the day of HCG injection.

The therapy of early ovarian pregnancy is surgical in the first place, and in the event that the patient desires a future pregnancy, the conservation of ovarian tissue is the clinical goal of the treatment. In our case, we could preserve the ovaries and she could conceive later and deliver.
